# Supplementary material for: Genomic loci associated with grain protein and mineral nutrients concentrations in Eragrostis tef under contrasting water regimes
Source: Front Plant Sci. 2024 Dec 20;15:1458408. doi: 10.3389/fpls.2024.1458408 (PMC11695128; doi:10.3389/fpls.2024.1458408)
Supplement: Supplementary file 1 [file DataSheet1.pdf]

Supplementary information

Genomic Loci Associated with Grain Protein and Mineral Nutrients concentrations in *Eragrostis tef* under Contrasting Water Regimes

Muluken Demelie Alemu[1,2], Shiran Ben-Zeev[1], Vered Barak[1], Yusuf Tutus[3], Ismail Cakmak[3], Yehoshua Saranga[1]*

[1]R. H. Smith Faculty of Agriculture, Food and Environment, The Hebrew University of Jerusalem, Rehovot, Israel

[2]Ethiopian Institute of Agricultural Research, Addis Ababa, Ethiopia

[3] Faculty of Engineering and Natural Sciences, Sabanci University, Istanbul, Turkey

*** Correspondence:**

shuki.saranga@mail.huji.ac.il

Table S1. ANOVA for the effects of treatment (well-watered [WW] and water-limited [WL]), seed color (SC) and genotype (nested within SC) on tef grain nutrient concentrations under field conditions in 2021.

**Source**	**df**	**Prot (%)**	**Fe (mg/kg)**	**Cu (mg/kg)**	**Mn (mg/kg)**	**Zn (mg/kg)**	**Ca (%)**	**K (%)**	**Mg (%)**	**P (%)**	**S (%)**
Treatment	1	119.84 ***	20.36***	47.28***	802.94***	6.39*	107.27***	3.6ns	158.1***	55.89***	249.36***
Genotype [SC]	1	73.73 ***	3.42***	4.83***	4.28***	5.23***	1.93***	1.95***	4.05***	4.32***	5.03***
SC	175 (221)	6.82 ***	0.03ns	83.88***	277.06***	25.07***	21.1***	19.43***	7.67**	7.54**	27.81***
Treatment*SC	1	2.93									
Treatment*Genotype [SC]	175	1.41 **									
**Treatment effect**											
WW		12.00 b	38.00 b	8.56 b	46.55 a	35.56 b	0.191 a	0.499 a	0.191 a	0.431 a	0.205 b
WL		12.56 a	39.56 a	9.03 a	34.85 b	36.14 a	0.172 b	0.493 a	0.181 b	0.418 b	0.222 a
WL/WW ratio		1.05	1.045	1.055	0.75	1.02	0.9	0.99	0.95	0.97	1.08
**SC effect**											
White		12.50 a	38.75 a	9.11 a	37.26 b	36.43 a	0.177 b	0.503 a	0.185 b	0.422 b	0.216 a
Brown		12.06 b	38.81 a	8.48 b	44.14 a	35.28 b	0.186 a	0.489 b	0.187 a	0.427 a	0.211 b
Brown/White ratio		0.96	1	0.93	1.18	0.97	1.05	0.97	1.01	1.01	0.98

A full factorial nested model was used for the replicated protein (Prot) content, whereas a two-way ANOVA model was used for the non-replicated (bulked) mineral analyses (Fe, Cu, Mn, Zn, Ca, K, Mg, P, S). Degree of freedom (df) for the genotype effect was 175 for Prot (due to missing values) and 221 for the minerals. *, **, *** and ns indicate significant F-values at $p < 0.05$, 0.01, 0.001 and non-significant values, respectively. Different letter (a, b) indicates difference between values according to Student's t-test at $p < 0.05$. Relative effects of irrigation regime and SC are indicated below the mean values.

'Table S2. Pearson's correlation coefficients (r) between tef grain nutrient concentrations under well-watered (WW, lower part) and water-limited (WL, upper part) treatments in 2021.

	Prot	Fe	Cu	Mn	Zn	Ca	K	Mg	P	S	GY	TSW
**Prot**		0.54***	0.29***	-0.13ns	0.42***	0.12ns	0.05ns	0.19**	0.3***	0.49***	-0.54***	0.03ns
**Fe**	0.55**		0.34***	0.24***	0.63***	0.18*	0.01ns	0.39***	0.5***	0.26***	-0.35***	0.2**
**Cu**	0.23***	0.3***		-0.18**	0.37***	-0.08ns	0.29***	0.29***	0.34***	0.43***	-0.01ns	-0.03ns
**Mn**	0.12ns	0.32***	-0.09ns		0.21**	0.3***	-0.06ns	0.33***	0.34***	-0.1ns	0.13*	-0.1ns
**Zn**	0.5***	0.64***	0.38***	0.17*		0.04ns	0.18**	0.34***	0.58***	0.36***	-0.31***	-0.04ns
**Ca**	0.23***	0.22**	-0.12ns	0.31***	0.03ns		-0.08ns	0.31***	0.16*	0.09ns	-0.19**	-0.178*
**K**	-0.02ns	-0.13ns	0.27***	-0.09ns	0.02ns	0.01ns		0.25***	0.42***	0.25***	-0.02ns	0.03ns
**Mg**	0.39***	0.44***	0.24***	0.28***	0.37***	0.33***	0.17*		0.58***	0.35***	0.13*	-0.1ns
**P**	0.41***	0.49***	0.19**	0.38***	0.44***	0.25***	0.26***	0.64***		0.36***	-0.13ns	-0.04ns
**S**	0.5***	0.36***	0.31***	-0.03ns	0.48***	0.15*	0.19**	0.34***	0.44***		-0.26***	-0.1ns
**GY**	-0.28***	-0.21**	0.07ns	-0.04ns	-0.21**	-0.22**	0.46***	-0.04ns	0.08ns	-0.14*		-0.06
**TSW**	0.05ns	0.14*	-0.04ns	0.0ns	-0.08ns	-0.11ns	-0.1ns	-0.16*	-0.23***	-0.18**	-0.09ns	

Prot, protein; GY, grain yield; TSW, thousand seed weight. *, **, *** and ns indicate significant correlation at $p < 0.05$, 0.01, 0.001 and non-significant, respectively. Cell colors highlight significant positive (green), significant negative (red), non-significant (yellow)and auto (white) correlation .

Table S3. Single-nucleotide polymorphisms (SNPs) associated with candidate genes which may affect grain nutrient concentrations in tef under contrasting water regimes.

**Traits**	**SNP**	**Candidate gene**	Gene postion	Accession number	**CandidateGene function**	**Refence**
P (WW & WL)	1B_15526648	PTI1-like tyrosine-protein kinase 1	15,568,594-15,569,192	XM_002446667	pti1-like tyrosine-protein kinase important in disease resistance signaling mechanism	Dang et al., 2019
Fe (WW & WL)	1B_15704188	cell division control protein 48 homolog E-like	15,698,306-15,704,156	XM_025937909	Plays a crucial role in protein quality control and regulation of immune responses in plants. This helps maintain proper protein homeostasis during immune responses, contributing to the regulation of defense mechanisms against pathogen attacks	Copeland et al., 2016
Cu (WW & WL)	5A_6882876	polygalacturonase inhibitor 1-like	6,881,104-6,882,120	XM_034722340	ubiquitous plant cell wall proteins that are directed against fungal polygalacturonases (PGs), which are important pathogenicity factors	De Lorenzo and Ferrari, 2002
PC (WW & WL)	5A_7173734	ADP-ribosylation factor GTPase-activating protein AGD	7,152,409-7,156,513	XM_025944243	ADP ribosylation factor (ARF) -GTPases are important proteins that control membrane trafficking events	Stefano et al., 2010
Zn (WL) & K (WW)	6B_2255059	cytochrome b-c1 complex subunit 6	2,251,074-2,254,009	XM_002450285	essential component of the electron transport chain in mitochondria	Smith et al., 2012

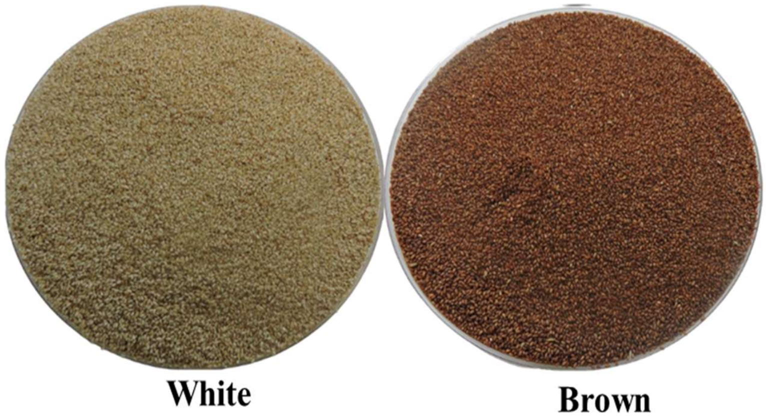

Figure S1. Tef diversity panel (TDP-300) grain colors visually scored into two broad categories: white and brown.

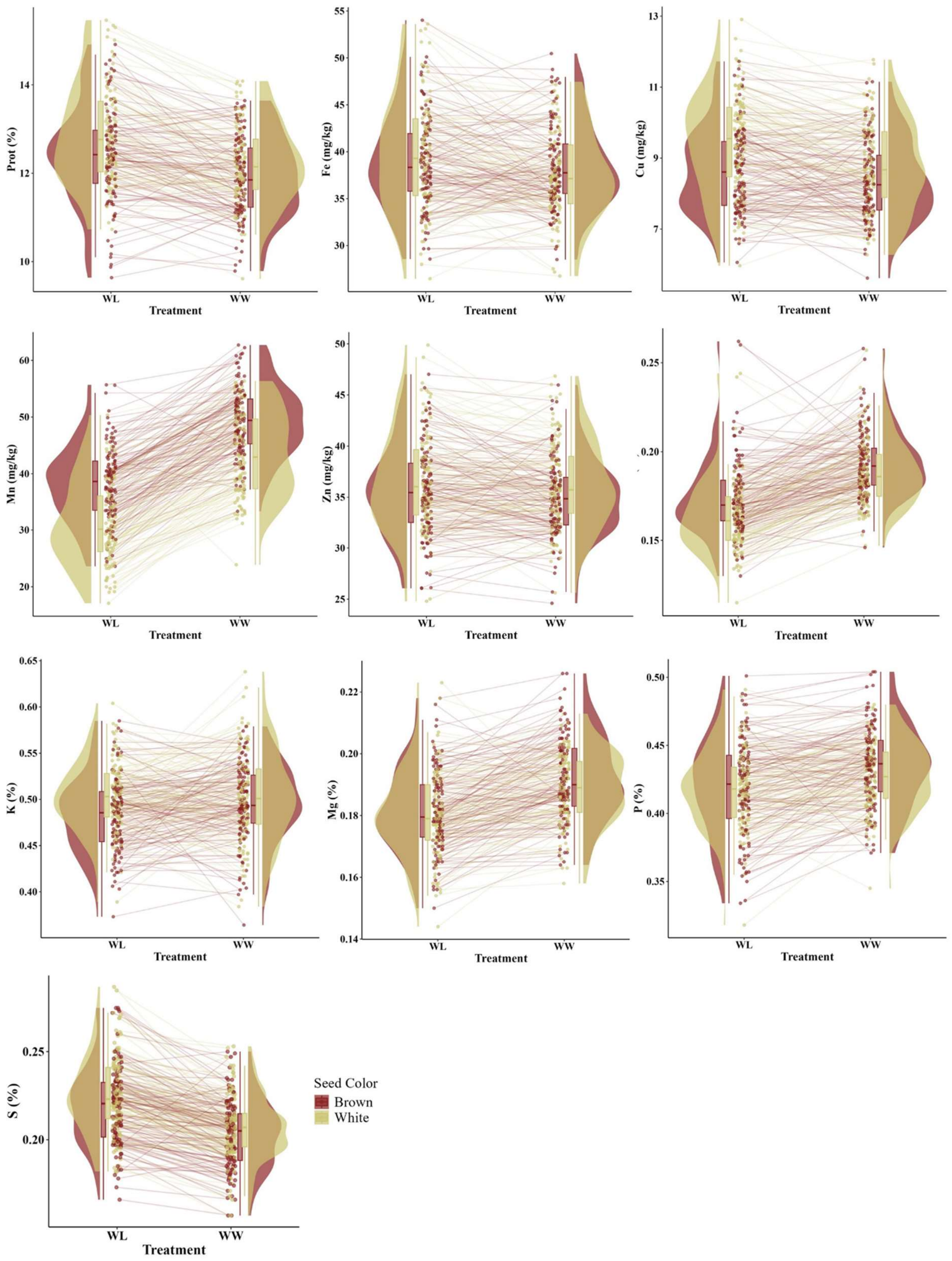

Figure S2. Raincloud plots depicting the effect of genotype (brown and white seed colors)-by-environment (well-watered, WW; water-limited, WL) interaction on the concentrations of tef grain nutrients in 2021. Prot, protein.

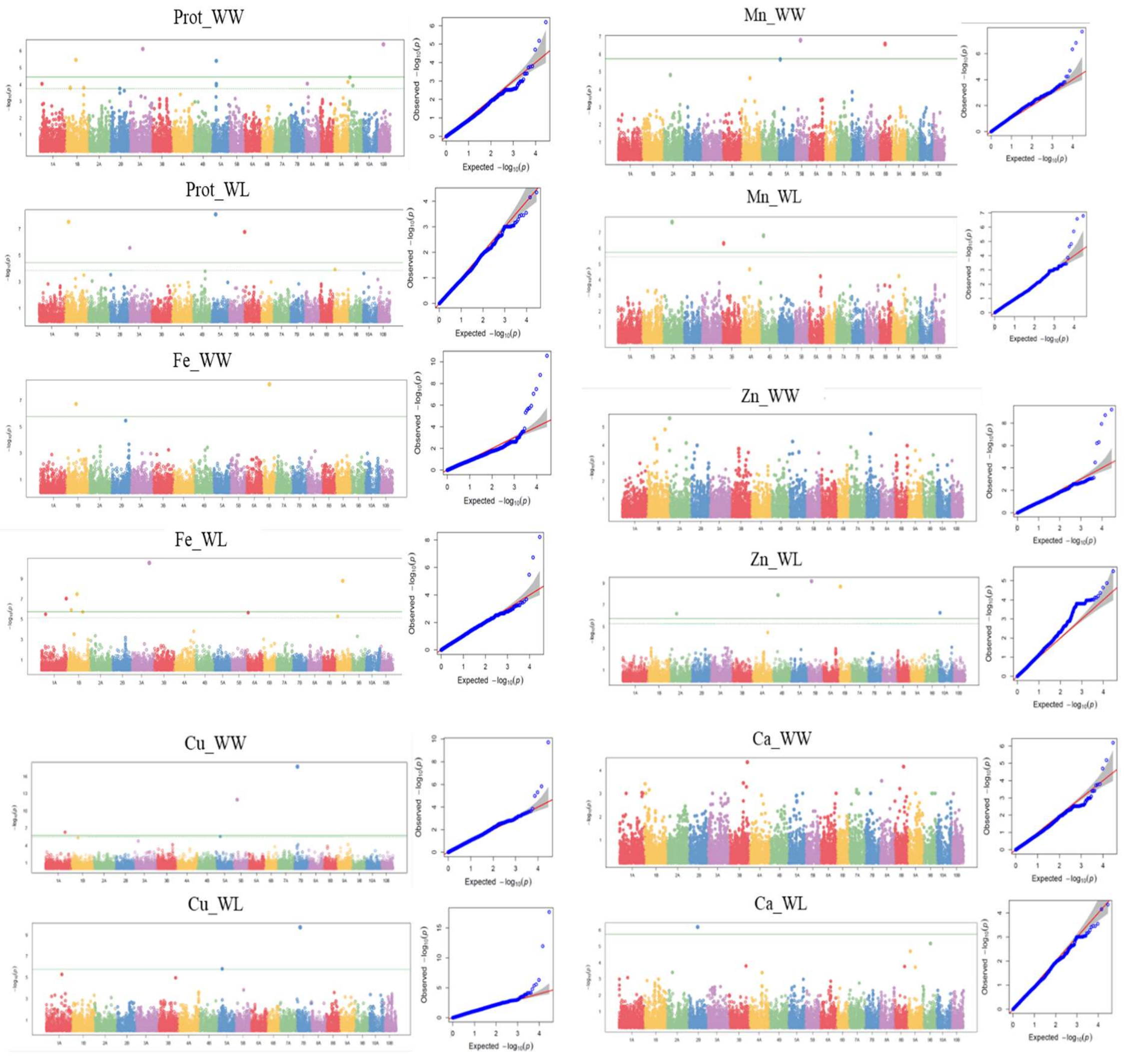

Figure S3. Genome-wide association study (GWAS) in tef. Manhattan plot showing 20 chromosomes with significant single-nucleotide polymorphisms (SNPs) associated with grain nutritional content and quantile-quantile (Q-Q) plot under well-watered (WW) and water-limited (WL) treatments in 2021. Prot, protein. Bonferroni threshold, $p < 1.7\text{E-}06$ (horizontal solid green line); less stringent threshold, $p < 1\text{E-}04$ (dashed green line). X-axis represents the 20 tef chromosomes.

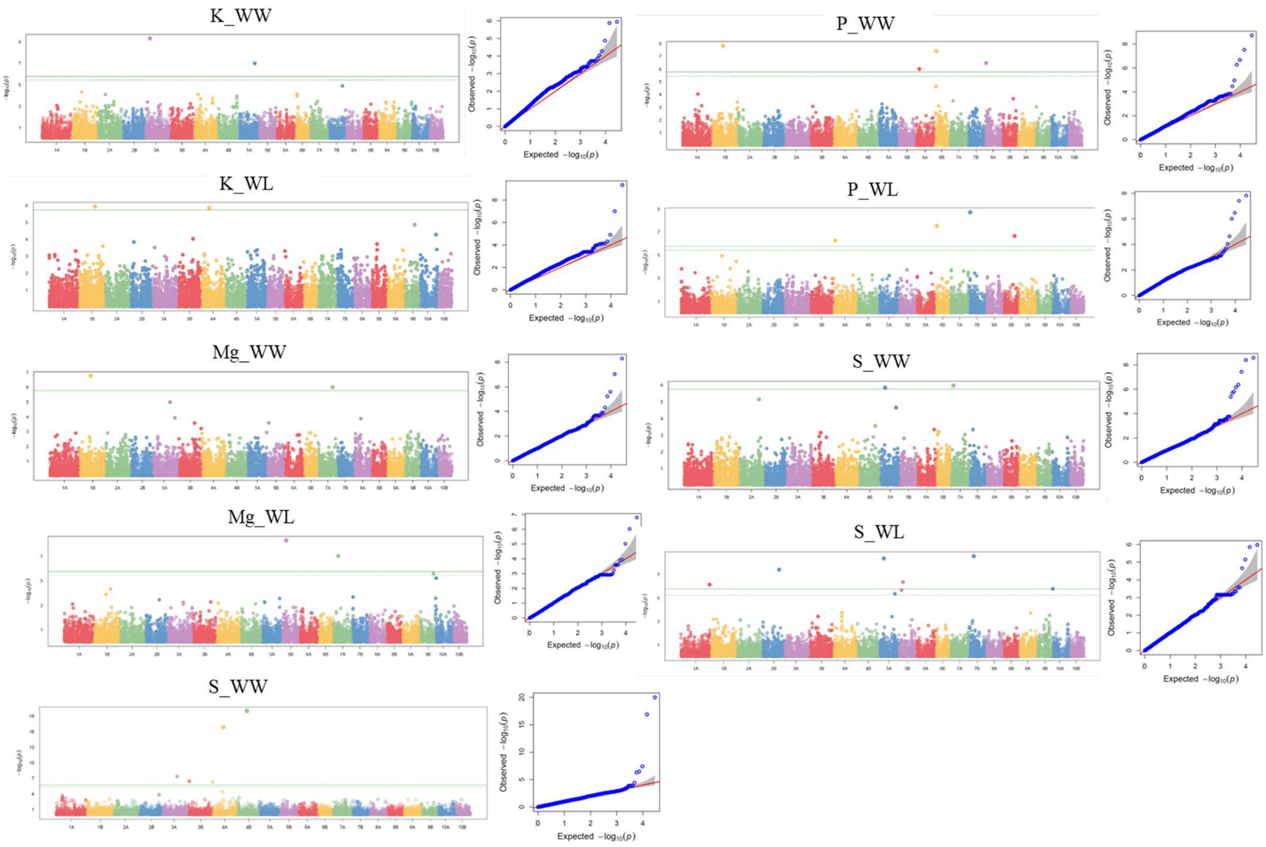

Figure S3. *Continued….*

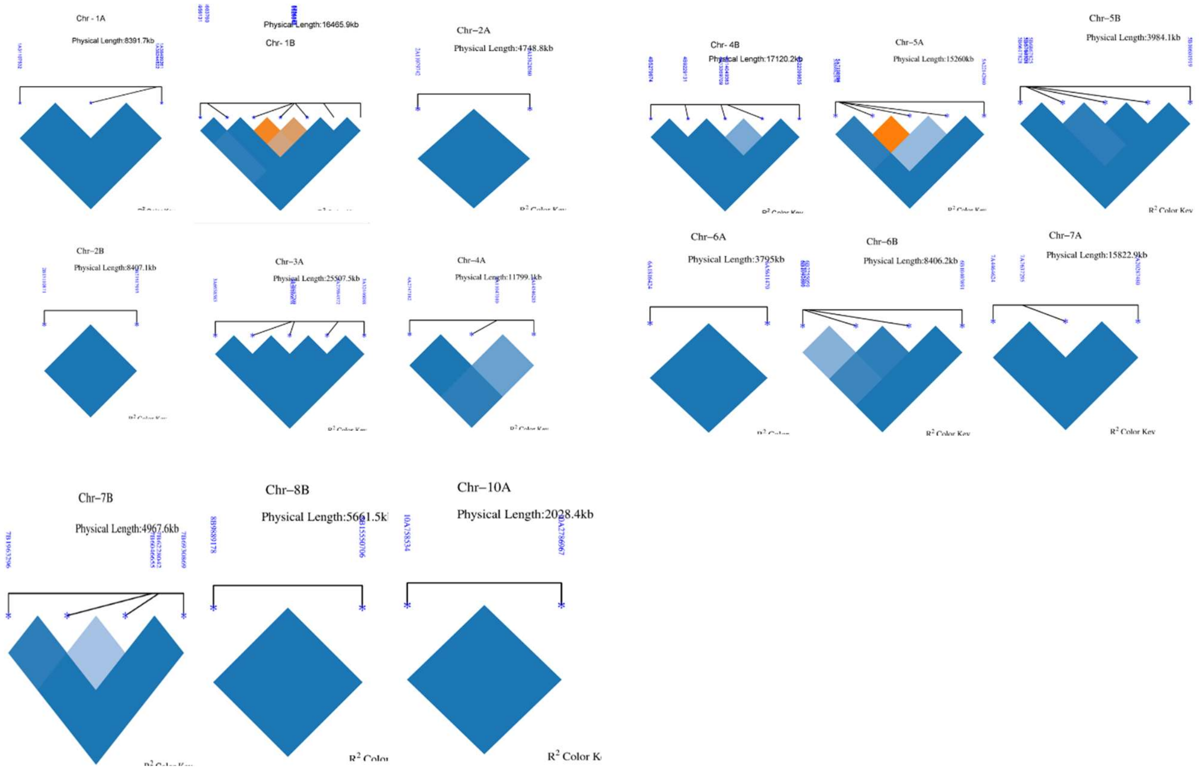

Figure S4. Graphical display of pairwise linkage disequilibrium (LD) heat map of 59 significant single-nucleotide polymorphisms (SNPs) on 15 out of 20 chromosomes (Chr) (excluding Chr 3B, 8A, 9A & B and 10B which exhibited single significant SNP).
